# Hypoplastic Left Heart Syndrome: Signaling & Molecular Perspectives, and the Road Ahead

**DOI:** 10.3390/ijms242015249

**Published:** 2023-10-17

**Authors:** Sayantap Datta, Wangjia Cao, Mikayla Skillman, Mingfu Wu

**Affiliations:** Department of Pharmacological and Pharmaceutical Sciences, College of Pharmacy, University of Houston, Houston, TX 77204, USA; sdatta20@cogarnet.uh.edu (S.D.); wcao9@cougarnet.uh.edu (W.C.); mskillma@cougarnet.uh.edu (M.S.)

**Keywords:** HLHS, cardiomyocytes, signaling mechanisms

## Abstract

Hypoplastic left heart syndrome (HLHS) is a lethal congenital heart disease (CHD) affecting 8–25 per 100,000 neonates globally. Clinical interventions, primarily surgical, have improved the life expectancy of the affected subjects substantially over the years. However, the etiological basis of HLHS remains fundamentally unclear to this day. Based upon the existing paradigm of studies, HLHS exhibits a multifactorial mode of etiology mediated by a complicated course of genetic and signaling cascade. This review presents a detailed outline of the HLHS phenotype, the prenatal and postnatal risks, and the signaling and molecular mechanisms driving HLHS pathogenesis. The review discusses the potential limitations and future perspectives of studies that can be undertaken to address the existing scientific gap. Mechanistic studies to explain HLHS etiology will potentially elucidate novel druggable targets and empower the development of therapeutic regimens against HLHS in the future.

## 1. Introduction

**Figure 1:**
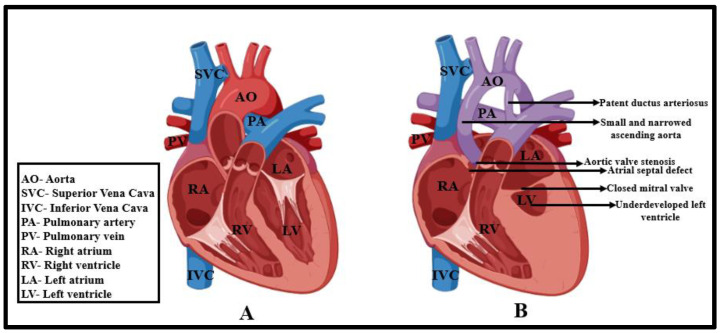
Schematic representation (cross-sectional view) of the physiological defects in a HLHS heart (**B**) as compared to that of a normal heart (**A**)—chiefly identified by patent ductus arteriosus, narrowed ascending aorta, defects in mitral valve and atrial septation, and a prominently compromised left ventricle and all the pictures were generated via BioRender [7].

Constituting approximately 1–2% of all known cardiovascular complications, HLHS roughly affects 8–25 per 100,000 neonates born globally without selection during pregnancy. In the United States of America alone, it affects approximately 1 out of every 3841 babies [1,2]. Phenotypically, HLHS is a condition characterized by atresia or stenosis of the aortic and mitral valves, hypoplasia of the left ventricle, and a narrowed ascending aorta (Figure 1) [3,4,5,6,7,8]. This accounts for the hypertrophy of the right atrium and leads to the delivery of unaerated blood to the lungs [6]. From an anatomical perspective, three major HLHS types are recognized: mitral and aortic stenosis, mitral stenosis and aortic atresia, and mitral and aortic atresia [9,10]. Over the years, studies utilizing human-induced pluripotent stem cells (hiPSCs) from HLHS patients, along with advancements in mouse genetic models, have identified an intrinsic issue in cardiomyocytes as a potential cause of HLHS, which is characterized by a decrease in cardiac differentiation efficiency, disorganized sarcomeres, abnormal mitochondrial structure, and impaired NOTCH signaling [11,12,13]. Furthermore, studies suggest that autosomal recessive inheritance and abnormalities in endocardium development could potentially contribute to ventricular and valvular hypoplasia in HLHS [8,14,15]. In fact, correlations between HLHS and left-sided lesions of the bicuspid aortic valve (BAV) and aortic coarctation have been based on epidemiology studies that identified BAV in first-degree relatives of HLHS probands [16].

The pathological changes of HLHS include the emergence of void spaces in the myocardium, vascular channel formations, and mononuclear cellular infiltration along the ventricular septum [17]. The void spaces that extend from the top of the ventricular septum into the lumen of the right ventricle are lined by mesothelial cells [18]. These spaces do not consist of blood cells and are potentially fistulas between the coronary artery and the right ventricular lumen [18]. These pathological changes hinder the right ventricle’s ability to function [6,19]. The abnormal development of the foramen ovale is another speculated pathological hallmark of HLHS. Its oblique orientation hinders the flow of blood from the right atrium [6,20]. This abnormally developed foramen ovale potentially accounts for irregular blood flow from the inferior vena cava to the left atrium at the fetal stage [20]. Although comprehensive studies have characterized HLHS phenotypic features throughout the years, understanding the molecular basis of HLHS incidence remains largely obscure to date. Gaining deeper insights into the molecular basis of HLHS will lead to a significant understanding of HLHS etiology. This review focuses on the potential molecular mechanisms outlining HLHS that are instrumental in manifesting one or more of the above-mentioned phenotypic features. A clear understanding of the molecular mechanisms will potentially enable the identification of drug targets to develop novel therapeutic strategies for minimizing risk factors and improving HLHS treatment over the long term.

## 2. Prenatal and Postnatal Risks

A fetus with HLHS usually remains stable, with cases relating to in utero demise being rare and mainly governed by chromosomal abnormalities [21]. The right ventricle remains functional in HLHS-affected fetal hearts, with propensities to detect HLHS being likely at around the 20-week gestational period [22,23]. Close examination of pulmonary venous return and blood flow patterns across the atrial septum has elicited potentially significant restrictions around the atrial septum [24]. Under these circumstances, blood egress from the left auricle and pulmonary venous circulation exhibits a high propensity, thus encountering hindrance [24], leading to pulmonary vasculopathy and severely damaged lungs. These fetuses are at an enormously high risk due to the potential of the pulmonary vasculature being underdeveloped, leading to poor oxygenation [25].

After birth, the pulmonary vascular resistivity is high, and the ductus arteriosus persists. However, the pulmonary resistance decreases shortly, and a high amount of blood becomes shunted across the pulmonary vascular bed. This significantly compromises systemic circulation [26], resulting in tachypnoea, hypotension, and acidosis [27]. The systemic perfusion becomes hampered even more when the ductus arteriosus starts closing. These undiagnosed individuals usually exhibit feeding and respiratory difficulties a few hours after birth or at 2–3 days, eventually culminating in shock and cardiac failure [26,27].

## 3. Existing Surgical Perspectives

### 3.1. Stage I—Norwood Procedure

**Figure 2:**
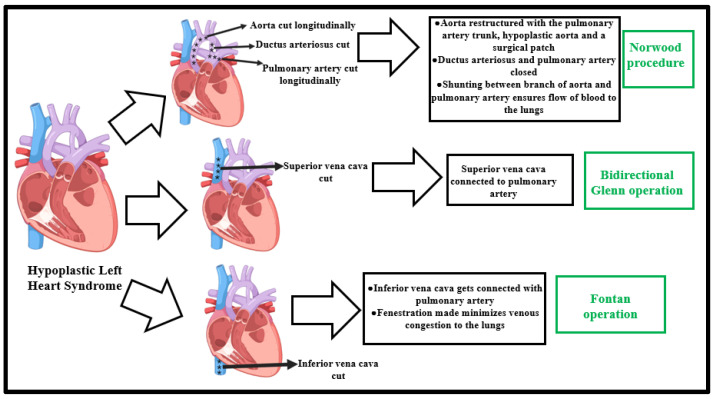
Schematic representation of existing surgical perspectives for HLHS treatment. The Norwood procedure is aimed at increasing systemic oxygen delivery and oxygen perfusion levels; the Glenn operation separates the systemic and pulmonary venous blood flow by directing blood returning from the superior vena cava to directly enter the pulmonary circulation; and the Fontan operation connects the inferior vena cava with the pulmonary arteries and completes the separation of the systemic and pulmonary circulation via an extracardiac conduit [7].

The first stage of surgery in HLHS treatment is the Norwood procedure, usually carried out within the first week after birth [28]. It is aimed at increasing systemic oxygen delivery and organ perfusion levels by reconstructing the aorta and developing a connection between the aorta and the right ventricle (Figure 2) [29]. This assures sufficient intracardiac mixing through the atrial communication pathway, coupled with a restrictive blood supply to the lungs through the developed shunt [28,29].

### 3.2. Interstage Period

This is the phase between stage I and stage II, which is associated with a significant amount of risk with regard to morbidity, growth failure, and mortality [30]. More than 10–15% of the mortalities associated with HLHS occur during this phase [30,31]. It is characterized by what gets clinically identified as “red flag symptoms”—increased fussiness, diarrhea, and vomiting; poor extent of feeding; change in skin pigmentation; reduced oxygenation levels; increased sleepiness, etc. [31].

Given such conditions, the adoption of single-ventricle inter-stage monitoring programs (ISVMPs) and their standardization by the National Pediatric Cardiology Quality Improvement Collaborative have reduced fatal consequences in the inter-stage period [31]. Such monitoring programs usually include biweekly hospital visits with echocardiograms, alternating with biweekly follow-ups with the pediatrician for assessment of the homeostasis of other parameters [32].

### 3.3. Stage II—Superior Cavopulmonary Connection Establishment

Widely known as the bidirectional Glenn operation, it occurs between 3 and 6 months of age [33]. This is aimed at separating the systemic and pulmonary venous blood flow by allowing blood returning from the superior vena cava (SVC) to enter directly into the pulmonary circulation. It is achieved by transecting the SVC prior to its insertion into the right atrium and establishing a connection between the SVC and the pulmonary artery [34]. In contrast to the Norwood procedure, this step is associated with a higher percentage of survival among HLHS populations (approximately 93%) [27]. The infants start exhibiting improved cardiac activity levels after this stage, especially because of an improvement in physiological reserve [35]. The oxygen saturation level shoots up to about 80–90% [27,35]. However, the development of the lower body with age and increased oxygen utilization bring about cyanosis. This demands the intervention of the stage III step of treatment [35].

### 3.4. Stage III—Fontan Operation

Outlining the concluding step of HLHS surgical treatment, this step is carried out between 18 months and 4 years of age [36]. Here, the inferior vena cava (IVC) gets connected to the pulmonary arteries in completion of the separation between the systemic and pulmonary circulations [36]. This is achieved via an extracardiac conduit, whereby a conduit tube-graft (of 16–20 mm in diameter) is connected from the IVC to the right pulmonary artery [36,37]. This step also offers a small residual right-to-left shunt and culminates in oxygen saturation levels between 90 and 95% [37]. In fact, the efficient placement of fenestration enables a decrease in venous congestion and optimized oxygen delivery. This fenestration also improves stroke volume, besides optimizing oxygen delivery [38]. Long-term survival possibilities with an unaffected Fontan circulation are encouraging, predicting survival rates to be around 94% after the first year, 90% after 10 years, 85% after 15 years, and 74% after 20 years of Fontan operation [39].

## 4. Signaling and Molecular Mechanisms Outlining HLHS Incidence

### 4.1. Endocardial-Related Signaling Pathways

Endocardial cells are specialized endothelial cells outlining the innermost layer of the heart wall [40]. Besides serving as the source of mesenchymal cells in the endocardial region that give rise to structural elements of the atrioventricular valves, endocardial cells also account for the maturation and development of the atrial and membranous ventricular septa [41].

**Figure 3:**
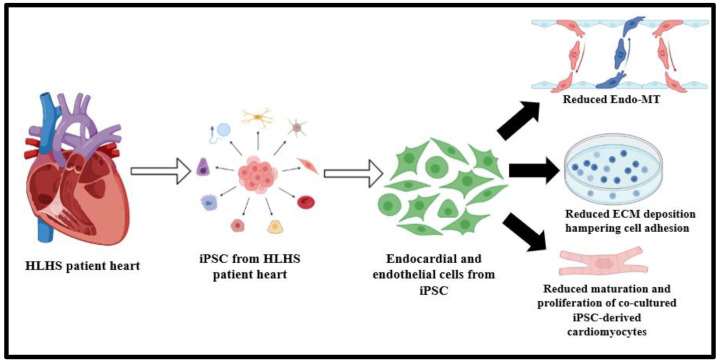
Schematic representation of endocardial dysfunctions characterizing HLHS, characterized by a decrease in Endo-MT transition, compromised ECM deposition, and a decrease in maturation and proliferative capacity of cardiomyocytes [7].

Extracellular matrix (ECM) deposition is important for initiating trabeculation and its subsequent maturation [42,43]. The endocardial ridges are intrinsically rich in hyaluronic acid and fibronectin content, which promotes the rate and capacity of cardiomyocyte proliferation with increased myocardium mass [8,44,45,46]. Previous studies have shown augmented deposition of ECM-rich fibrous tissue via endothelial-to-mesenchymal transition (Endo-MT) [47]. The excessive fibroblasts in the endocardial fibroelastosis (EFE)-associated tissues are mainly epicardium-derived [42,48]. Investigating the molecular mechanisms governing the endocardial defects in HLHS, single-cell RNA (scRNA) sequencing studies with iPSC-derived endocardial cells (iECs) of HLHS hearts illustrate that anomalous ECM deposition and Endo-MT in endocardial as well as endothelial cells lead to a decrease in proliferation and maturation of cardiomyocytes, thus characterizing early stage HLHS (Figure 3) [7,49]. Reduction in blood flow, tissue hypoxia, and other environmental factors in the later stages ensure that EFE occurs because of the involvement of both epicardium and endocardium-derived fibroblasts [48,50,51]. De novo mutations of genes (*TFE3*, *EDNRA*, *ZNF292*, *FOXM1*, *ZMYND19*, *PCBP3*, *TCF12*, *ARID1B*, *NOVA1*, *PKD1*, *RBFOX2*, *ST5*, *TSC1*, *USP8*, *HERC4*, *KMT2D*, *ETS1*, *CHD7*, *CTR9*, *GLA*, *FMNL1*, *PHRF1*, *SIPA1L1*, and *HIRA*) predominantly manifest in the endocardium, coronary, and lymphatic ECs, providing a consolidated basis for targeting the endocardium in HLHS pathogenesis [8]. These studies demonstrate functional abnormalities through the impairment of ECM deposition, Endo-MT, and vascular endothelial growth factor receptor (VEGF) signaling, which are all functionally important pathways that play a pivotal role in valve formation and cardiac remodeling [42,52].

### 4.2. Notch Signaling

**Figure 4:**
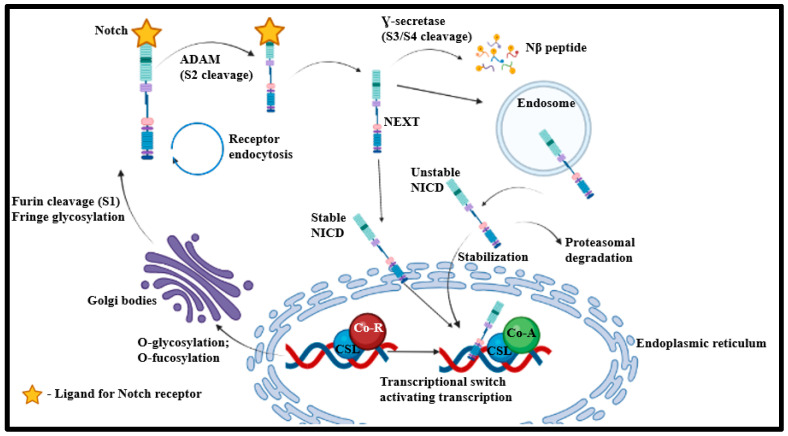
Schematic representation of the NOTCH signaling cascade. Followed by proteolytic cleavage by Furin at site 1 (S1), the mature Notch receptor produced is activated by the neighboring cell-presented ligand. The endocytosis of the ligand-receptor enables conformational alterations in the bound Notch receptor and presents S2 for ADAM metalloprotease-based cleavage. This leads to a Notch extracellular truncated (NEXT) fragment that is acted upon by Ɣ-secretase. Ɣ-secretase cleaves the transmembrane fraction of NEXT from S3 to S4 and releases NICD and Nβ-peptide. Further cleavage at the membranous domain enables the generation of stable NICD. This stabilized NICD then undergoes nuclear translocation and then associates with the DNA-binding CSL protein. CSL otherwise associates with a ubiquitous co-repressor (Co-R) and histone deacetylases. Post-NICD association, allosteric changes in CSL displace the corepressor complex, enable transcriptional coactivator masterminds to mark out the NICD-CSL interface, and eventually recruit co-activator A (Co-A) for subsequent transcriptional cascades [7].

Notch signaling is evolutionarily a conserved cascade that is vital for cell–cell communication, tissue boundary maintenance, cellular fate determination, renewal, and differentiation of stem cells (Figure 4) [7,13,53,54,55,56,57,58,59]. Biphasic Notch modulation plays a pivotal role in differentiating murine embryonic stem cells (mESC) and iPSC, considering that Notch signaling activation augments mesodermal induction in early-stage differentiation [60]. However, such an event hinders cardiac progenitor formation in the later stages of differentiation. The activation of Notch1 and Notch4 signaling in mESC-derived haemangioblasts is responsible for the specification of their cardiac fate. This demonstrates that Notch signaling functionality is diverse based on the target cells and their temporal differentiation index [13,61]. NOTCH1 is associated with HLHS incidence primarily because of its pivotal role in Mendelian calcific aortic valve disease and compound heterozygous NOTCH1 mutations in HLHS subjects [62,63,64]. Analytical studies with HLHS-iPSC-derived cardiomyocytes exhibit notable downregulation in the expression of NOTCH1, NOTCH2, NOTCH3, and NOTCH4. A similar extent of downregulation is also exhibited by NOTCH target genes such as *DTX1*, *FOX*, *HEY2*, and *HEYL* [13,59]. This shows that the NOTCH signaling pathway is significantly hindered in HLHS conditions.

Additionally, downregulation is exhibited by NOTCH-binding proteins like JAG1 and JAG2, pointing out an autocrine feedback loop in NOTCH signaling [13]. Examining HLHS-specific hiPSCs also exhibits the downregulation of NOTCH signaling, which is mediated through the nitric oxide (NO) signaling process [65]. This stems from the idea that Notch signaling mediates valve formation by inducing Activin A, which in turn stimulates NO in endothelial cells that exhibit Endo-MT [55,66]. This additionally augments NO cell surface receptors. NO plays a pivotal role in the differentiation and specification of mESCs within ectodermal and mesodermal lineages, thus improving the cardiomyocyte yield of differentiating mESCs [67]. A minute investigation of differentiating HLHS-hiPSCs indicates differences in NO levels in the overall cell population, which highlights patterning and cell fate specification cascades of Notch signaling [11]. HLHS-hiPSCs with the highest NO levels also exhibit elevated levels of Notch Intracellular Domain (NICD), thus establishing the association between Notch signaling and NO generation [11]. Through the use of markers for early mesoderm or endodermal cells (CXCR4) [68], it was revealed that cells producing higher NO levels were indeed CXCR4+. These findings suggest that HLHS-hiPSC reduces the NO signaling cascade and hinders the cardiomyocyte yield from the early progenitor cell population. This hypothesis gains further basis with NO supplementation by eliciting increased cyclic guanosine monophosphate (cGMP) levels and activating the NO-cGMP-protein kinase G(PKG) cascade [11].

Studies advocating differentiation of HLHS iPSCs to cardiomyocytes using a Jagged peptide (Notch ligand) show that mutations in the Notch4 peptide domain potentially affect the cytoplasmic expression of Notch proteins and inhibit proteasomal activity [69]. In turn, this impairs Notch protein-associated functional cascades. However, these mutations do not impact Jagged binding to NOTCH4 and subsequent NOTCH signaling activation in HLHS-patient cardiomyocytes [13]. In theory, this suggests the druggability of Notch signaling, whereby activation of Notch signaling can potentially restore Ca^2+^ homeostasis in HLHS iPSC-derived cardiomyocytes. However, such a hypothesis’s molecular and functional basis needs validation for further consolidation.

### 4.3. TGF-β/BMP Signaling

The transforming growth factor (TGF)-β/bone morphogenetic protein (BMP) signaling plays a vital role in cardiac developmental processes and associated disease conditions [70]. The manipulation of this signaling pathway primarily results in altered cardiomyocyte proliferation, differentiation, and associated growth cascades [71,72,73,74]. Along these lines, expression profiles of TGF-β-associated genes were compared between HLHS right ventricle (HLHS-RV) samples and their control counterparts [70]. Such comparative analyses show that HLHS-RV samples exhibit increased levels of activin receptor type IIA (ACVR2A) and activin receptor-like kinase 1 (ACVRL1). Both activin receptor type IIA and activin receptor-like kinase 1 are largely involved in tissue remodeling [75,76]. Other significantly upregulated genes associated with the TGF-β signaling cascade include *CDC25A*, *p21*, *p15*, *BMP5*, *BMP3*, *GDF3*, *NODAL*, and BMP binding endothelial regulator (*BMPER*). All of these genes play pivotal roles in cellular survival, growth, and differentiation [70]. Additionally, significant alterations are observed in levels of anti-mullerian hormone receptor 2 and the BMP antagonist Inhibin alpha. These findings suggest that HLHS-RV genes can be potential players associated with myocardial remodeling, growth, and differentiation. In fact, significantly increased levels of ACVR2A and ACVRL1 can potentially culminate in compensatory changes in hemodynamic pressure, myocardial remodeling, and tissue repair in HLHS-RV tissue.

### 4.4. Wnt/SHH/p53 Signaling

**Figure 5:**
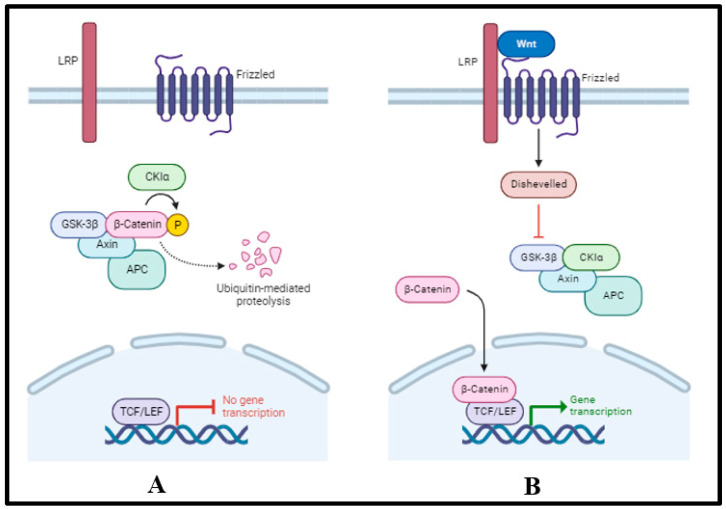
Schematic representation of (**A**) inactivated and (**B**) activated Wnt signaling pathways. In the absence of the Wnt ligand, phosphorylation of β-catenin occurs and leads to complexation with axin, adenomatous polyposis coli (APC), and glycogen synthase kinase (GSK)-3-β. This complex is subsequently subjected to proteasomal degradation. In the presence of the Wnt ligand, β-catenin remains unphosphorylated and enters the nucleus to drive transcription [7].

The Wnt signaling is known to exert a bidirectional impact across different stages of cardiomyogenesis (Figure 5) [77]. It is activated during the development of the early embryo in the lateral plate mesoderm and is inhibited to ensure that the heart eventually develops to its proper size [78]. Through amalgamating whole genome sequencing, iPSC technology, and model validation with a familial approach, studies over the years have endeavored to elucidate novel HLHS-associated genes and explain the underlying mechanisms involved [79]. These studies have led to the identification of lipoprotein-related protein 2 (LRP2, also known as megalin) as being involved in HLHS pathogenesis. LRP2 is a multi-ligand endocytic receptor expressed in a multitude of tissue sites, but primarily in absorptive epithelial tissues. It is a glycoprotein with an extracellular binding domain, a single transmembrane domain, and a short carboxy-terminal cytoplasmic tail [80]. The extracellular domains are responsible for binding albumin, apolipoprotein B, apolipoprotein E, and lipoprotein lipase [81]. Functionally, LRP2 plays a vital role in the reuptake of lipoproteins, sterols, and hormones and in cell signaling [82,83].

Furthermore, LRP2 is a chief modulator of cardiomyocyte proliferation, maturation, and development, which has been substantiated in *Drosophila* and zebrafish models [79]. Studies reveal that missense mutations in *LRP2* are more frequent in HLHS patients than in their control counterparts. The deleterious mutations could potentially manifest the HLHS phenotype through the Wnt, SHH, and p53 signaling pathways [79]. iPSC studies have shown that p53 depends on Lrp2 expression, and *Lrp2* mutations account for anomalies in the p53 pathway that lead to the ventricular hypoplasia characteristic of HLHS [84]. Alongside LRP2, hypomorphic variants of *Trol*/*HSPG2* and *Apolpp*/*APOB* potentially alter Wnt and sonic hedgehog (SHH) signaling [85] and initiate HLHS. However, genetic interaction-associated studies need to be carried out to elucidate the mechanistic role even further [86].

### 4.5. Can Single Gene Mutation Attribute to HLHS?

#### 4.5.1. RBFOX2

RNA-binding Fox-1 Homolog 2 (RBFOX2) belongs to a family of RNA-binding proteins with a strong affinity for (U)GCAUG-rich sequences highly conserved in vertebrates [87,88]. It is known to regulate alternative splicing cascades in embryonic stem cells, pluripotent cellular differentiation, and epithelial–mesenchymal transition [89,90,91]. The existing body of literature suggests that conditional ablation of *Rbfox2* is associated with developmental abnormalities [92]. In fact, knockdown of *Rbfox2* and its paralog *Rbfox1* reduces heart rate and attributes to myofibrillar disarray [93]. Murine model studies over the years have shown that *Rbfox2* downregulation correlates with pressure overload and, subsequently, heart failure [94,95,96].

Recent studies have pointed out the potential role of *Rbfox2* in HLHS pathogenesis. HLHS-specific mutations in *RBFOX2* result in abnormalities associated with RBFOX2 expression [89]. HLHS-specific splice-site mutations include 1.6 kb intron 10 and degradation of RBFOX2 mRNA by nonsense-mediated decay (NMD) [89]. HLHS-specific frameshift mutation incorporates a stop codon and culminates in NMD. Similarly, the HLHS-based nonsense Rbfox2 mutation deletes a part of the C-terminal domain (CTD) of the Rbfox2 protein [89,97]. This CTD is otherwise important for mediating Rbfox2 interaction with other RNA-binding proteins, spliceosome component U1C, and nuclear localization [97,98,99]. HLHS-specific *RBFOX2* mutations manipulate the cellular and subcellular localization of Rbfox2 in HLHS patients [100]. Although RBFOX2 exhibits nuclear and cytoplasmic localization in the right ventricle of control subjects, RBFOX2 levels become severely downregulated in the right ventricular cardiomyocytes of HLHS patients [89]. This affects RBFOX2 functionality regarding RNA metabolism and attributes to transcriptomic alterations in HLHS patients. From a pathological perspective, RBFOX2 has also been identified as being responsible for gene expression alterations in the right ventricle of HLHS patients [101]. Cross-linking immunoprecipitation followed by RNA-sequencing (CLIP-seq) studies integrated with transcriptomic data from HLHS patients show that *RBFOX2* mutations lead to cardiac transcriptome alterations in HLHS patients by mRNA dysregulation of genes involved in cell cycle and metabolism [89]. Such genes chiefly include *Pnn*, which encodes the Pinin protein and regulates epithelial cellular differentiation [102], *Phkb*, which encodes for glycogen phosphorylase kinase and enables cellular growth [103], *Ddx39,* which is an RNA helicase and modulates the interplay between proliferation and differentiation of cells, and *Mcm7*, which is essential for DNA replication and cellular growth [104].

#### 4.5.2. SAP130

Sin3-associated protein 130 (Sap130) is a subunit of the histone deacetylase-dependent SIN3A corepressor complex msin3A. Sap130 can enable the assemblage and enzymatic activity of msin3A to ensure interactions between the sin3A corepressor complex and other regulatory complexes [105].

*Sap130* manipulations and their correlation with HLHS incidence have been established utilizing CRISPR-Cas9 gene editing [12]. CRISPR mouse lines also exhibit germline transmission of *Sap130* alleles and express the spliced Sap130 transcript with an in-frame deletion of 36 amino acids [12]. This truncation is also exhibited by *Ohia* mutant mice lineages [106]. Peri-implantation lethality phenotypes in *Sap130* knockout (homozygous and heterozygous) mice substantiate the hypomorphic nature of *Sap130*. In fact, *Sap130a* antisense-morpholino-mediated knockdown in the zebrafish model shows reduced ventricular cardiomyocyte count and a shortened ventricle 72 h after fertilization [12]. This is when the heart consists only of the first heart field derivatives, similar to murine left ventricle progenitors [107].

#### 4.5.3. PCDHA9

ProtocadherinA9 (*PCDHA9*) is a member of the protocadherin-α gene cluster [108]. This protocadherin-α gene cluster consists of cadherin superfamily genes with highly similar and related coding sequences [109]. The array of N-terminal variable exons is followed downstream by constant C-terminal exons [110]. The large N-terminal exons encode six cadherin ectodomains, and the C-terminal exons encode the cytoplasmic domain [111]. The encoded cadherin-like cell adhesion proteins are important plasma membrane proteins that are significant in maintaining cellular connectivity [112].

*Pcdha9* mutations are attributed to aortic hypoplasia, stenosis, cardiac hypertrophy, bicuspid aortic valves, hypoplastic left ventricle, and mitral valve—a spectrum comprising primary as well as secondary phenotypic features associated with HLHS [12]. In fact, CRISPR mice lineages exhibit germline transmission of doubly targeted *Pcdha9* alleles [12]. This chiefly refers to an in-frame amino acid insertion that deletes two adjacent amino acids near the *Ohia Pcdha9* missense mutation site. Functional studies have also pointed out that this mutation is characteristically loss-of-function in correlation with HLHS onset. However, most *Pcdha9* mutation-associated HLHS phenotypes are more pronounced with a simultaneous *Sap130* mutation, pointing towards some type of synergism between *Sap130* and *Pcdha9* mutations in triggering HLHS incidence [12].

#### 4.5.4. CONNEXIN43

The gap junction channels are formed by CONNEXIN43 proteins, which play a significant role in developmental processes through the direct cellular exchange of signaling molecules [113]. Studies over the years have pointed out that CONNEXIN43 channels are gated by phosphorylation, and intervention with this regulation results in cardiac laterality defects and malformations in humans, chickens, and frogs, culminating in HLHS [114,115]. However, deeper insights into the mutational studies have shown that *CONNEXIN43* mutations constitute a minor population of *CONNEXIN43* alleles [113]. Such mutational patterns are typically of the same kind: two silent polymorphisms and two missense mutations whereby arginine at positions 362 and 376 is replaced by glutamines [113]. In vitro and in vivo protein kinase A and protein kinase C-mediated phosphorylation studies point out that the substitution of arginine residues at 362 and 376 positions diminishes phosphorylation in the regulatory domain of *Connexin43*, underlying a potential mechanism governing HLHS pathogenesis [116,117]. Such findings potentially point towards the idea that HLHS incidence in fetal developmental stages may potentially occur owing to interventions in one or more of the signaling pathways that utilize *Connexin43* governing left heart formation [118,119,120].

#### 4.5.5. HAND1

Heart and neural crest derivatives-expressed protein 1 (*HAND1*) is a member of class B basic helix-loop-helix (bHLH) transcription factors [121]. This bHLH domain carries DNA binding and dimerization motifs that consist of basic amino acid chains, an amphipathic α-helix, a loop, and an additional α-helix [122,123]. HAND1 heterodimerizes with class A E-factors like TCF3 (E2A, E12/E47) and with closely related Hand2 [122]. Studies also show that HAND1 can potentially activate or suppress transcription [62,124]. This depends on the target sequence (consensus E-box or degenerate Thing1/D-box) and the dimerization partner [125]. Physiologically, *Hand1* is chiefly involved in placentation, dorsoventral patterning, interventricular septum formation, and cardiac morphogenesis in the embryonic heart [126,127,128].

In determining whether HAND1 exhibits any role in HLHS incidence, a typical frameshift mutation has been identified whereby a G nucleotide at the 376 position gets deleted [121]. This affects the amino acid sequence in the bHLH domain of Alanine 126 (A126). In fact, this mutation was found to be highly frequent among HLHS patients [126]. This A126 frameshift (A126fs) mutation, amidst all the other infrequent nonsynonymous alterations induced, is a dominant predictor mutation characterizing the HLHS condition. It results in prematurely truncated protein expression at amino acid 137 [121]. This consists of a 12-amino acid segment, which is a typical characteristic of the mutant protein and potentially contains limited α-helical content. The studies elucidating the mobility and expression of Hand1 showed remarkably reduced expression of mutant protein amounts, potentially because of reduced mRNA stability [121].

#### 4.5.6. Myrf

Multiple studies reported that point mutations of myelin regulatory factor (*MYRF*) are associated with the occurrence of HLHS [129,130,131], suggesting the possibility that monogenic mutations can cause HLHS. Myrf is a vital membrane-bound transcription factor involved in the development of the urogenital, neural, visual, and cardiac systems [132,133,134,135,136]. Myrf consists of two essential fragments: the N-terminal and C-terminal portions. The N-terminal fragment comprises a transactivation domain and a DNA-binding domain [137]. The C-terminal domain contains an intramolecular chaperone auto-processing (ICA) domain and an endoplasmic reticulum luminal domain [138]. The ICA domain plays a pivotal role in triggering the homo-trimerization of Myrf [135]. Following the trimerization, Myrf undergoes an automatic cleavage process, and the N-terminal portion translocates into the nucleus [135,139,140]. This translocation is a fundamental step for Myrf to function effectively as a transcription factor.

Numerous studies have reported that patients carrying de novo variants in *MYRF* exhibit a variety of CHDs. Notably, among these CHD cases, the most prevalent form was HLHS, accounting for 44% of the cases, while scimitar syndrome followed as the second common CHD, constituting 31% [131]. According to the published data, the individuals diagnosed with HLHS exhibit de novo mutations in both the N-terminal and C-terminal segments of the MYRF protein, indicating a complex underlying mechanism. The association between *Myrf* and HLHS has been further investigated using CRISPR-Cas9 technology in the vertebrate medaka model, and the *Myrf* mutant line exhibited a significantly prominent hypoplastic ventricle, which closely recapitulates the phenotypes observed in pediatric patients [129]. Overall, the signaling mechanism by which *Myrf* contributes to the pathogenesis of HLHS remains unclear, and there is limited knowledge in this area. This underscores the pressing need for further extensive research in this area.

## 5. Limitations in Current Understanding of HLHS Etiology

Over the years, the clinical studies associated with HLHS have primarily been centered around surgical procedures and heart transplantation in mitigating HLHS [139]. Although the surgical mode of treatment provides hope for the survival of infants born with HLHS, the consequences in the long run are yet to be well defined [141]. Several issues surrounding surgical treatment remain poorly defined to date, and the long-term outcomes and quality of life post-surgery remain poorly understood.

The heritability of HLHS is a widely accepted hypothesis because of the multitude of genes and their corresponding mutations bearing a direct correlation with HLHS occurrence and consequent phenotypic characterization. However, the majority of the heritability studies regarding HLHS lack HLHS-phenotypic specificity. This is because most of these studies focus on phenotypic outcomes like left ventricular hypoplasia, which is not a feature of HLHS alone but rather a diverse variety of cardiovascular malformations that include a disturbed atrioventricular septal defect. From a mechanistic point of view, different courses of mutations have been elucidated in a multitude of genes. Although such mutations are correlated with the HLHS phenotype, the detailed idea regarding the mechanistic flow empowering HLHS onset largely remains obscure to date. This stems from the lack of a suitable animal model beyond the digenic *Sap130* and *Pcdha9* mutant models [12], primarily restricting the understanding of genetic mutations and HLHS onset and keeping the findings chiefly restricted to transcriptomic and in vitro studies. These studies additionally demand the exploration of a greater expansion of genes promising novel mechanistic possibilities and their role in HLHS incidence, further substantiating the heritability studies.

In view of the signaling pathways that play important roles in HLHS onset, existing paradigms of studies have established the correlation between such cascades and the HLHS phenotype. However, a detailed understanding of the molecular basis of such cascades needs to be investigated to establish the correlation further. Most of these studies are currently restricted to the iPSC model of analysis. This assumes great challenges since iPSC differentiation might not completely mimic the endocardium or cardiomyocytes and their unique properties. The signaling pathways usually exhibit complicated courses of crosstalk with other signaling pathways in mediating their actions [142]. The absence of such understanding currently with respect to HLHS onset limits the idea of having consolidated inferences until further downstream signaling events are explored [42]. In fact, the potential of the iPSC-derived endothelial and endocardial cells to mature into valve interstitial cells (VIC) and valve endothelial cells (VEC) remains exploratory to date, thus restricting the understanding of valvular defects and VIC-VEC crosstalk in HLHS pathogenesis [143,144,145,146]. Beyond the known genetic and signaling processes responsible for HLHS onset, other factors via maternal pregestational or gestational diabetes also have an association with HLHS onset. The genetic and non-genetic environmental basis that characterizes HLHS etiology needs to be understood and explored further.

## 6. Future Perspectives

Ongoing studies in the characterization of iPSC-differentiated valvular cells by endocardial and endothelial markers, viz., NPR3, CDH11, NFATC1, and TGF-β2, shall empower further investigation of VEC and VIC function with respect to HLHS onset [52,147,148,149]. The development of reliable HLHS-specific mice models, outlining one of the major focuses of our lab, can potentially enable a better pathological understanding of HLHS. Ongoing studies have also shown that fibroblast growth factor 8 (FGF8) is important for mesodermal cell fate determination and Wnt activation for Endo-MT in cardiac progenitor cells [42]. The iPSC-derived epicardial cells can potentially differentiate into endocardial cells. These cell lineage tracing studies may provide more of an idea of the developmental origin of endocardial cells and elucidate novel mechanisms regarding HLHS incidence [148].

Cardiac organoids constitute another emerging platform to comprehend cellular communications during heart development. In fact, the current paradigm of studies has shown that myocardial defects such as cardiac hypertrophy and myocardial infarction can be mimicked by the cardiac organoid model, which exhibits anatomical patterns resembling in vivo models undertaken to date [150]. Further investigations along these lines can enable a better understanding of endocardium–myocardium crosstalk and the underlying molecular basis that can better highlight the endocardial and myocardium-associated dysfunctions in HLHS [151]. Advocating human germline genome editing can be another potential direction towards developing HLHS therapy [152]. However, different cells of the embryo are modified differently owing to the persistence of CRISPR/Cas9 activity even after the zygote begins to divide [153]. Hence, optimization of these factors and the potential pediatric stage for application need to be contemplated to better understand the implications of this otherwise novel line of therapy.

## 7. Conclusions

In summary, existing courses of study point towards a multifactorial etiological basis for HLHS. Surgical and transplantation modes of treatment constitute currently existing tools for ameliorating HLHS, but the extent of success achieved remains highly debatable. By gaining a deeper insight into the cellular and molecular mechanisms governing HLHS pathogenesis, these strategies will be significantly instrumental in the identification of potential druggable targets and the development of novel therapeutic strategies for treating this CHD in the following years.

## Data Availability

Not applicable.
